# Surgeon General’s Fever Circular

**Published:** 1878-10-20

**Authors:** 


Surg. Gen'l's Fever Circular.—Surgeon-General Woodworth has issued a circular to physicians and others residing at points visited by yellow fever, announcing the organization of the Commission to gather and record facts relative to the commencement and spread of the epidemic, and asking the cordial co-operation of all who have facts to communicate upon that subject. A preliminary report of the facts gathered up to the 19th proximo will be presented to the American Public Health Association, which will convene in Richmond on that date to discuss the report.

